# Platelet Count to Prothrombin Time: A Noninvasive Predictor of Esophageal Varices in Patients With Chronic Liver Disease

**DOI:** 10.7759/cureus.59627

**Published:** 2024-05-04

**Authors:** Syed Rohail Ahmed Rizvi, Muhammad Danish Ashraf Wallam, Arif Rasheed Siddiqui, Syed Afzal Ul Haq Haqqi, Zea Ul Islam Farrukh, Saad Khalid Niaz, Muhammad Umar Farooq, Fahad Kakar, Atif A Hashmi

**Affiliations:** 1 Gastroenterology, Patel Hospital, Karachi, PAK; 2 Pathology, Liaquat National Hospital and Medical College, Karachi, PAK

**Keywords:** platelet count to prothrombin time ratio, prothrombin time, platelet count, esophageal varices, chronic liver disease

## Abstract

Introduction

Esophageal variceal bleeding is a potentially deadly consequence of portal hypertension in patients with cirrhosis. Although upper gastrointestinal endoscopy is still the preferred method for identifying esophageal varices (EV), the present study measured the platelet count to prothrombin time (PLT/PT) ratio for the assessment of portal hypertension and subsequent diagnosis of EVs in patients with chronic liver disease (CLD).

Methods

This was an observational comparative study conducted in the outpatient department of Patel Hospital, Karachi, Pakistan, using a non-probability consecutive sampling technique. Ethical approval was obtained from the Patel Hospital ethical review committee (PH/IRB/2022/028). An independent sample t-test was used for parametric data, whereas the Mann-Whitney U test was used for non-parametric data. The chi-square test was used to compare the categorical data of patients with and without EV. Receiver operating characteristic (ROC) analysis was performed to evaluate the cutoff values for the PLT/PT ratio, sensitivity, specificity, and area under the curve (AUC).

Results

The study involved 105 patients with and without EV. Among them, 38 (63.3%) males and 22 (36.7%) females had EV, whereas 30 (66.7%) males and 15 (33.3%) females did not. The platelet (PLT) count was also significantly lower in patients with EV (87.6 ± 59.8) than in those without (176.6 ± 87.7) (p < 0.001). The PLT/PT ratio was significantly lower in patients with EV (median: 5.04, IQR: 3.12-9.21) compared to those without (median: 14.57, IQR: 8.08-20.58) (p < 0.001). The sensitivity and specificity of the PLT/PT ratio for identifying EVs were 97.80% and 83.30%, respectively.

Conclusion

We found a significantly lower PLT/PT ratio in cases with EV than those without EV. After defining an optimal cutoff, PLT/PT had a high sensitivity in identifying cases with EVs in CLD. Therefore, we conclude that in patients with CLD, the PLT/PT ratio is a noninvasive predictor for the presence of EV.

## Introduction

Worldwide, cirrhosis is the leading cause of morbidity and mortality. It was the 11th most common cause of death and the 15th most common cause of morbidity worldwide in 2016, accounting for 2.2% of fatalities and 1.5% of disability-adjusted life years [1]. In 2017, 1.32 million people died from chronic liver disease (CLD), with roughly two-thirds of the deaths occurring in males and one-third in females [2]. Liver cirrhosis leads to portal hypertension and associated complications, which are characterized by degeneration of liver cells, tissue scarring, and mixed regenerating nodules. Pakistan has the highest incidence of CLD among Asian nations [3].

In clinical terms, portal hypertension is described as a pathological increase in portal vein pressure resulting from various causes, the most common being schistosomiasis in Africa and liver cirrhosis in Western countries [4,5]. Esophageal and gastric varices can cause fatal bleeding from portal hypertension in patients with liver cirrhosis [6]. Although less frequent, gastric variceal bleeding is more complicated than esophageal variceal bleeding [7]. Approximately 50% of patients with cirrhosis have esophageal varices (EV), whereas 20% of patients with cirrhosis have gastric varices, either by themselves or in conjunction with EV [8,9].

The causes of portal hypertension, which can result in EV, include massive splenomegaly, obstruction of the portal vein, alpha-1 antitrypsin insufficiency and primary biliary cirrhosis, constrictive pericarditis, chronic right-sided heart failure, and Budd-Chiari syndrome [10]. Bleeding is frequently observed near the gastroesophageal junction because varices have the most fragile walls and are most superficial there. Approximately 50% of cases of acute variceal hemorrhage stop spontaneously [11].

The earliest and least expensive way to diagnose portal hypertension and its complications is through the clinical evaluation of EV using medical history and clinical assessment. The existence of EV is directly associated with splenomegaly, ascites, hepatic encephalopathy, and a diminutive liver. Patients with compensated cirrhosis, however, may have EV [12].

The most effective method for EV screening in patients with cirrhosis is still esophagogastroduodenoscopy (EGD), which allows direct visualization of EV and risk indicators. Although it is an invasive method, an objective, repeatable, accurate, and noninvasive technique should be used to diagnose EV [13].

Studies have investigated the application of a variety of radiological modalities, such as MRI, contrast-enhanced MRI, barium, conventional ultrasonography, Doppler ultrasound, splenoportography, angiography, endoscopic ultrasound, computed tomography, computed tomography with contrast enhancement, and FibroScan, for the identification of EV [14]. Predicting EVs also involves noninvasive biochemical parameters. These included prothrombin time (PT), serum albumin, serum bilirubin, leukocyte count, platelet count (PLT), and hemoglobin. Currently, a number of ratios combining biochemical and radiological parameters have been established to predict the presence of EV [15].

Because there are not enough endoscopes or endoscopists in government hospitals in developing nations with limited resources, it is impossible to screen every cirrhotic patient to grade their EV and choose which patients should receive preventive therapy. Finding dependable and noninvasive indicators is required to identify the EV grades in Pakistan’s population. For endoscopists to prioritize endoscopic procedures on patients who are more likely to bleed, we must find noninvasive and trustworthy indicators to evaluate the EV grades in our population. Therefore, the present study evaluated the PLT/PT ratio for envisaging the existence of EV in patients with CLD.

## Materials and methods

Patient selection

This was an observational comparative study performed in the outpatient department of Patel Hospital, Karachi, Pakistan, using a non-probability consecutive sampling method. Ethical approval was obtained from the Patel Hospital Ethical Review Committee (PH/IRB/2022/028). The study period was six months. Consent of each patient has been given in writing. The study included 105 patients in the age range of 30-55 years, both male and female, who were diagnosed with liver cirrhosis. Patients on medication for lower portal hypertension, sclerotherapy recipients, hepatocellular carcinoma patients, gastrectomy patients, critically ill patients with liver cirrhosis, prior portosystemic anastomosis, and other factors contributing to ascites were not included in the study.

Clinical and biochemical parameters

Every patient underwent a thorough medical history, clinical evaluation, and laboratory tests to measure liver and renal function as well as total blood count, PLT, PT, and the international normalized ratio (INR). We used a fully automated chemistry analyzer to measure urea and creatinine. All laboratory tests were performed in the clinical pathology department of Patel Hospital. The PLT/PT ratios were computed and subjected to statistical analysis. Following screening for EGD, the patients were separated into two groups based on the presence and absence of EV.

Abdominal ultrasound and gastroesophageal endoscopy

All patients underwent abdominal ultrasound in the radiology department and upper gastroesophageal endoscopy for EVs screening in the endoscopy suite. Experienced radiologists and gastroenterologists performed the procedures. Both the sonologists and the endoscopists were blind to the laboratory and clinical parameters. The grading system used to classify EV was based on size; grade I included varices in the mucosa; grade II included varices not flattening with insufflation, not confluent, and occupying less than one-third of the esophageal lumen; grade III included large varices not flattening with insufflation and occupying more than a third of the esophageal lumen; and grade IV consisted of varices covering over two-thirds of the esophageal lumen [16].

Data analysis

Data were entered and analyzed using IBM SPSS Statistics, version 26.0 (IBM Corp., Armonk, NY). Descriptive statistics, such as quantitative variables, are reported as means and standard deviations, and frequencies and percentages are reported for qualitative variables. The Shapiro-Wilk test was used to check the normality of the data. For numerical variables, a comparison was seen between patients with and without EV; an independent sample t-test was used for parametric data, whereas a Mann-Whitney U test was used for nonparametric data. The chi-square test was used to compare the categorical data of patients with and without EV. A receiver operating characteristic (ROC) analysis was performed to evaluate the cutoff points for the PLT/PT ratio, sensitivity, specificity, and area under the curve (AUC). A p-value of 0.05 was considered statistically significant.

## Results

Clinical, biochemical, and endoscopic findings of patients with and without EV

The study involved 105 patients with and without EV. Among them, 38 (63.3%) males and 22 (36.7%) females had EV, whereas 30 (66.7%) males and 15 (33.3%) females did not, with no significant difference in gender distribution observed (p = 0.723). The mean age of patients with EV was 40.33 ± 15.98 years and without EV was 41.93 ± 12.72 years, indicating no significant difference (p = 0.899). Similarly, there was an insignificant association in body mass index (BMI) between the two groups (p = 0.131). Nevertheless, the etiology of cirrhosis exhibited significant variation between the groups (p = 0.029), with 31 (51.7%) patients with EV having hepatitis C virus as the primary cause. Patients with EV exhibit significantly higher incidences of irregularities in INR (p < 0.001) and lower albumin levels (p < 0.001) than those without EV. Moreover, the portal vein diameter was notably larger in patients with EV (p = 0.038). Furthermore, the PLT/PT ratio was significantly lower in patients with EV (median: 5.04, IQR: 3.12-9.21) compared to those without EV (median: 14.57, IQR: 8.08-20.58) (p < 0.001). Among patients with EV, 24 (40.0%) were categorized as Child-Pugh class A, 26 (43.3%) as class B, and 10 (16.7%) as class C. In contrast, among patients without EV, the majority belonged to Child-Pugh class A (36; 80.0%), with fewer in class B (9; 20.0%) and none in class C, with a statistically significant association among them (p < 0.001). EV grading also revealed significant differences in frequencies; the most common grading was I, followed by II, which was found in 26 (43.3%) and 27 (45.0%) patients, respectively (p < 0.001), as depicted in Table 1.

**Table 1:** The demographic characteristics, etiology, and grading of esophageal varices (n = 105) *p-value significant as <0.05. The data has been presented as n, %, mean ± SD/median (IQR). SD: standard deviation; BMI: body mass index; HBV: hepatitis B virus; HCV: hepatitis C virus; HDV: hepatitis D virus; INR: international normalized ratio; PLT/PT: platelet count to prothrombin time ratio; IQR: interquartile range

Variables	Patients with esophageal varices, n (%)/mean ± SD	Patients without esophageal varices, n (%)/mean ± SD	p-value
Gender			
Male	38 (63.3%)	30 (66.7%)	0.723
Female	22 (36.7%)	15 (33.3%)	
Age (years)	40.33 ± 15.98	41.93 ± 12.72	0.899
BMI (kg/m^2^)	22.26 ± 5.34	22.01 ± 4.25	0.131
Etiology of cirrhosis			
HBV	5 (8.3%)	13 (28.9%)	0.029*
HCV	31 (51.7%)	21 (46.7%)	
HBV+HDV	7 (11.7%)	6 (13.3%)	
Autoimmune	5 (8.3%)	2 (4.4%)	
Other	5 (8.3%)	3 (6.7%)	
Alcoholic	7 (11.7%)	0 (0.0%)	
INR	1.29 ± 0.20	1.13 ± 0.20	<0.001*
Albumin (mg/dL)	3.03 ± 0.687	3.64 ± 0.640	<0.001*
PLT/PT; median (IQR)	5.04 (3.12-9.21)	14.57 (8.08-20.58)	<0.001*
Portal vein diameter (cm)	1.12 ± 0.17	1.04 ± 0.18	0.038*
Child-Pugh class			
A	24 (40.0%)	36 (80.0%)	<0.001*
B	26 (43.3%)	9 (20.0%)	
C	10 (16.7%)	0 (0.0%)	
Esophageal varix grading			
Grade I	26 (43.3%)	0 (0.0%)	<0.001*
Grade II	27 (45.0%)	0 (0.0%)	
Grade III	6 (10.0%)	0 (0.0%)	
Grade IV	1 (1.7%)	0 (0.0%)	
Absent	0 (0.0%)	45 (100.0%)	

Differences in hematological parameters and renal and liver function tests in patients with and without EV

Patients with EV demonstrated lower mean hemoglobin levels (10.28 ± 2.08 g/dL) than those without EV (11.66 ± 2.04 g/dL), with a significant relationship among them (p = 0.001). Furthermore, total leukocyte count (TLC) was notably lower in patients with EV (4.47 ± 2.33) than in those without (6.52 ± 2.16), with a highly significant difference (p < 0.001). PLT was also significantly reduced in patients with EV (87.6 ± 59.8) compared with those without (176.6 ± 87.7) (p < 0.001). Additionally, significant differences were detected in PT, urea, creatinine, and gamma-glutamyl transferase (GGT) levels between the two groups (p < 0.001). Nonetheless, insignificant differences were observed in mean corpuscular volume (MCV), sodium levels, alkaline phosphatase (ALP), aspartate aminotransferase (AST), and alanine aminotransferase (ALT) between patients with and without EV, as depicted in Table 2.

**Table 2:** The comparison of liver and renal function tests and complete blood count between patients with and without esophageal varices *p-value significant as <0.05. The data has been presented as n, %/mean ± SD. SD: standard deviation; Hb: hemoglobin

Variables	Patients with esophageal varices, n (%)/mean ± SD	Patients without esophageal varices, n (%)/mean ± SD	p-value
Complete blood count			
Hb (g/dL)	10.28 ± 2.08	11.66 ± 2.04	0.001*
Total leukocyte count (×10^3^/m^3^)	4.47 ± 2.33	6.52 ± 2.16	<0.001*
Platelet count (×10^3^/mm^3^)	87.6 ± 59.8	176.6 ± 87.7	<0.001*
Mean corpuscular volume	81.2 ± 7.95	81.6 ± 7.53	0.789
Prothrombin time (sec)	14.31 ± 2.62	12.64 ± 2.97	<0.001*
Renal function test			
Urea (mg/dL)	25.5 ± 18.16	51.22 ± 53.19	<0.001*
Creatinine (mg/dL)	0.80 ± 0.64	2.26 ± 3.56	0.003*
Sodium (mEq/L)	133.6 ± 20.6	137.5 ± 4.91	0.738
Liver function test			
Alkaline phosphatase (IU/L)	213.5 ± 180.7	201.8 ± 193.7	0.534
Aspartate aminotransferase (IU/L)	86.03 ± 65.7	59.6 ± 41.1	0.134
Alanine transaminase (IU/L)	54.5 ± 33.7	49.9 ± 43.4	0.271
Gamma-glutamyl transferase (IU/L)	72.5 ± 70.9	120.0 ± 155.7	<0.001*

Sensitivity and specificity of PLT/PT ratio in identifying EV

**Figure 1:**
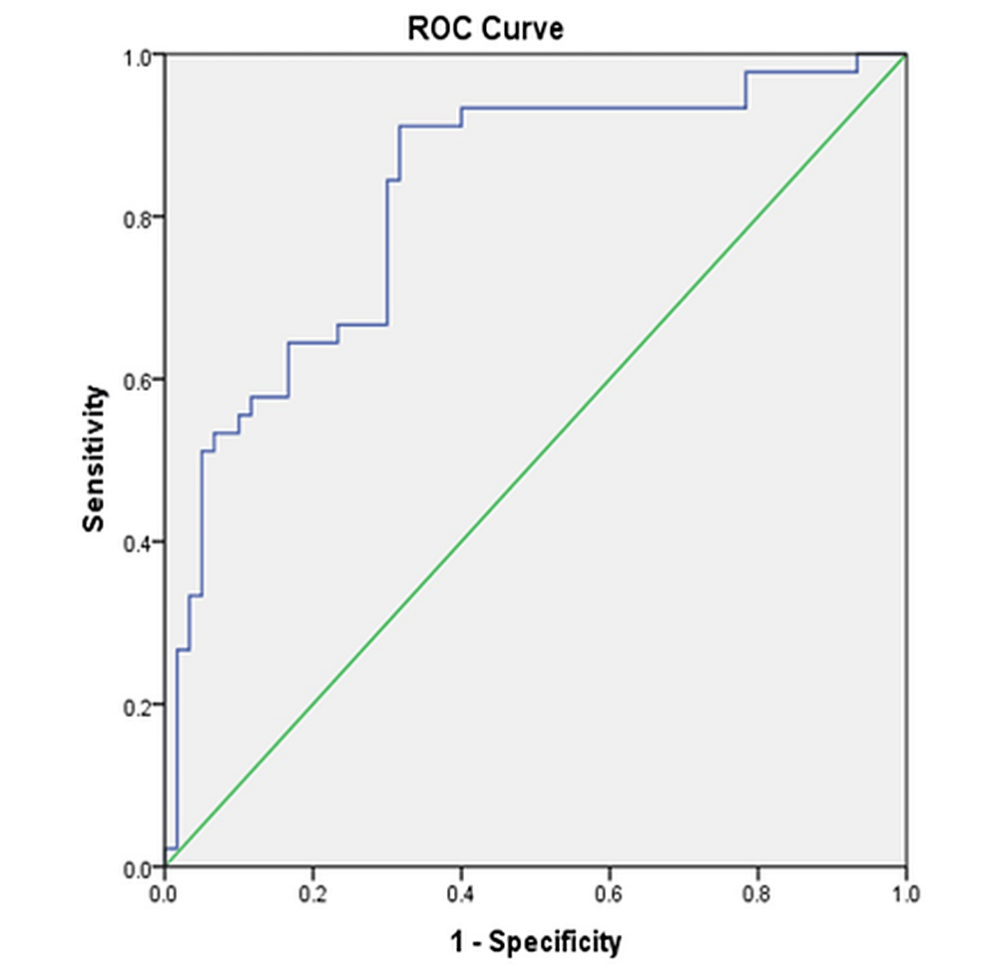
Receiver operator characteristic curve to determine the best cutoff values for platelet count/prothrombin time ratios in predicting the existence of esophageal varices

The receiver operator characteristic curve showed that the AUC was noted to be 0.823, showing good discriminative ability with an extremely substantial association (p < 0.001), suggesting that the PLT/PT ratio is a significant predictor of EV. The optimal cutoff value for the PLT/PT ratio was 2.5487. The sensitivity and specificity of the PLT/PT ratio for identifying EV were 97.80% and 83.30%, respectively, as shown in Table 3 and Figure 1.

**Table 3:** The cutoff value, sensitivity, and specificity of PLT/PT ratio *p-value significant as <0.05. The data has been presented as n%. AUC: area under the curve; PLT/PT: platelet count to prothrombin time ratio

	AUC	Confidence interval	p-value	Cutoff	Sensitivity	Specificity
PLT/PT	0.823	0.741-0.096	<0.0001*	2.5487	97.80%	83.30%

## Discussion

Several diagnostic tests are available to identify portal hypertension. Measuring portal pressure directly is an invasive process. Thus, it is preferable to perform a minimally invasive procedure, such as upper gastrointestinal tract endoscopy [17]. In patients with CLD, the ratio of PLT/PT may be significant for assessing portal hypertension and diagnosing EV, although upper gastrointestinal endoscopy (UGIE) is still the gold standard for diagnosing gastroesophageal varices.

A prospective study was conducted in Bangladesh to assess the effect of PT on EV initiation. A total of 60 patients suffering from liver cirrhosis were randomly divided into two groups: group I (30 patients with EV) and group II (30 patients without EV). Of the 60 patients, 11 were females and 49 were males, with a mean age of 37.11 ± 14.81 years. In 43 (71.7%) cases, the hepatitis B virus was mostly found in cirrhosis. Wilson’s illness affected one patient (1.6%), non-B and non-C affected 12 (20%), and four (6.6%) cases had hepatitis C virus (HCV). The longer plasma PT (over four seconds) was found to be positively correlated with EV, with a sensitivity of 56.67% and a specificity of 73.33% [17]. This study was consistent with the aforementioned study and indicated that most patients with and without EV were males. The mean age of patients with EV was 40.33 ±15.98 years and without EV was 41.93 ± 12.72 years. As far as the underlying cause is concerned, a higher percentage of patients (31; 51.7%) with EV have HCV.

Another retrospective analysis conducted in Pakistan elaborated on the frequency and characteristics of EV in patients with liver cirrhosis who underwent UGIE. Of the 2089 patients, 92.6% had EV and 7.45% did not. Grade I EV was found in 766 (39.5%) cases, grade II EV in 465 (24%) cases, and grade III EV in 703 (36.4%) cases. A total of 1331 (63.7%) male and 758 (36.3%) female liver cirrhosis patients underwent UGIE. In all, 89.1% of the female patients had EV, compared with 94.6% of the male patients. Males were substantially more likely to experience EV (p < 0.01). Significant differences were detected in the mean age of patients diagnosed with EV (51.25 ± 10.03 years) and those diagnosed with no EV (49.26 ± 11.11 years), with a significant difference among them (p = 0.019) [18]. Likewise, according to another study in Pakistan, 90.48% of patients with liver cirrhosis had EV [19]. In contrast, a study by Khan et al. [20] mentioned that grade III EV is the most common grade of EV (77%). Similarly, a study by Duah et al. [8] found no correlation between patients’ ages and the existence or nonexistence of EV (p = 0.197) [8]. The present study was partially similar to the abovementioned studies and reported that out of 105 patients with and without EV, 38 (63.3%) males and 22 (36.7%) females had EV. While 30 (66.7%) males and 15 (33.3%) females did not, no significant difference in gender distribution was found (p = 0.723). The mean age of patients with EV was 40.33 ± 15.98 years, compared to 41.93 ± 12.72 years in those without, showing no significant difference (p = 0.899). The most common EV grade was I, followed by II, which was found in 26 (43.3%) and 27 (45.0%), respectively.

Similarly, this cross-sectional descriptive study, conducted in Pakistan, involved 110 patients, of whom 49 (44.5%) were females and 61 (55.5%) were males. The patients’ mean age was 59.89 ± 9.01 years. Within the range of 50,000-99,000/uL in 29 (26.4%), 100,000-150000/uL in 14 (12.7%), and >150,000/uL in 28 (25.5%) patients, the PLT was less than 50,000/uL in 39 (35.5%) of patients. In all, 26 (23.6%) of the patients had grade I EV, while 27 (24.5%), 37 (33.6%), and 20 (18.2%) of the patients had grades II-IV EV, respectively [21]. These findings were consistent with the findings of a study performed in Taiwan, in which 71% of cirrhotic patients were males, showing male dominance [22]. These findings were corroborated by the present study and indicated that most of the patients with and without EV were males. The PLT count was also significantly reduced in patients with EV (87.6 ± 59.8) compared with those without (176.6 ± 87.7) (p < 0.001). Additionally, the most common EV grade was I, followed by II, which was found in 26 (43.3%) and 27 (45.0%), respectively.

Numerous previous studies predicted the grading of EV and the need for endoscopy in patients with cirrhosis using PLT in conjunction with additional noninvasive indicators like platelet count/spleen diameter ratio (PC/SD ratio) and AST to platelet ratio index (APRI) [23]. This study used the ratio of PLT/PT, which is readily available, noninvasive, affordable, and resource-efficient. It also requires no specialized knowledge.

Interestingly, another study assessed the diagnostic accuracy of noninvasive indicators of EV in patients with cirrhosis. According to that study, the ideal cutoff value for the PC/SD ratio was ≤818, and it showed a sensitivity and specificity of 92.05% and 60%, respectively (AUC: 0.835) [24]. A different Chinese study that employed PSDR <909 as a cutoff value also produced results with a 73% positive predictive value and an 88% negative predictive value [25]. A report from Egypt indicates that a 939.7 PC/SD optimal cutoff value yielded 100% sensitivity and 95.6% specificity, which is superior in terms of diagnostic accuracy [26]. The present study was partially similar to the above-cited studies and showed that the AUC was 0.823, indicating good discriminative ability. The p-value associated with the AUC was highly significant (p < 0.001), suggesting that the PLT/PT ratio is a significant predictor of EV. The sensitivity of the PLT/PT ratio for detecting EV was 97.80%, indicating its high accuracy in identifying true positive cases. The specificity of the PLT/PT ratio was 83.30%, demonstrating its capability to accurately recognize true negative cases.

Similarly, one of the studies compared the PLT/PT ratio with the PC/SD ratio and assessed their ability to evaluate the occurrence of EV in Egyptian patients with cirrhosis linked to ≤HCV. There were 99 patients with HCV-related liver cirrhosis, comprising 41 without EV and 58 with non-bleeding EV. Analyses of receiver operator characteristics showed that the PLT/PT ratio at cutoff ≤9419.3 (AUC: 0.936) was a more effective test for detecting the existence of EV than the PLT/SD ratio at cutoff ≤993.75 (AUC: 0.888). The PLT/PT ratio had higher sensitivity, specificity, PPV, and NPV (95.31, 88.57, 93.8, and 91.2% for PLT/PT vs. 89.06, 85.71, 91.9, and 81.1% for PLT/SD, respectively) [27]. The present study showed that the AUC was 0.823, and the p-value was highly significant (p < 0.001), suggesting that the PLT/PT ratio is a significant predictor of EV. The sensitivity of the PLT/PT ratio for detecting EV was 97.80%, indicating its high accuracy in identifying true positive cases, and its specificity was 83.30%.

Limitations

This study had a few limitations. It was a single-centered study with a small sample size, including only cirrhotic patients; while many studies have indicated that the PC/SD ratio is more accurate in envisaging the size and grading of varices, we only used PLT to assess the grading of varices. This study failed to identify signs of recent or imminent bleeding, such as cherry red spots. Future multicenter studies using the spleen size ratio, immediate bleeding signs, and a specific cause of cirrhosis are recommended. Further prospective studies with a sizable sample size are necessary to validate the results.

## Conclusions

In this study, we found that PLT/PT was significantly lower in patients with EVs with underlying CLD. After defining an optimal cutoff, we found that the PLT/PT value had a high sensitivity and specificity in identifying EVs. Hence, the findings predict that in patients with CLD, the PLT/PT ratio is a noninvasive indicator of the presence of EV. More research is necessary to develop a prediction score and investigate additional predictors and markers of esophagogastric variceal bleeding.
